# Notes and News

**Published:** 1899-11-11

**Authors:** 


### Notes and News.

(Collected and Communicated.)

The proposed amalgamation between the Medical Protection Society and the Medical Defence Union seems to have come to nothing, the Defence Union having declined the proposals made by the Protection Society.

The hospital ships "Trojan" and "Spartan" have arrived at Durban. The American ladies in London are collecting subscriptions for an American hospital ship. The committee rooms of the fund are at Walsingham House, Piccadilly.

The hospital train which the Central Red Cross Committee is sending to South Africa is being completed with the utmost speed. It is being built at Birmingham, and as many as 400 men are at work on it night and day. It is understood that Sir John Furley will take charge of it.

In view of the urgent appeals now being made for contributions to the Mansion House and other funds for the relief of sufferers by the war, it has been decided to postpone until next year the dinner announced to be held under the presidency of Field Marshal Lord Roberts, K.P., V.C., in aid of the funds of the Victoria Hospital for Children.

The vaccination war at Leicester still goes on its way. The question has become more general now, and has resolved itself upon the point as to whether vaccination officers can prosecute independently of the Guardians. The Local Government Board have decided to make it the duty of vaccination officers to take proceedings should the Guardians fail to give instructions, but the Anti-Vaccination League questions their power to do this under the Act.

Mr. Birkin, of Arnold, Nottinghamshire, has presented a house named Forest House, standing in a park, to the committee of the Nottingham Children's Hospital. The committee desired to build a new hospital on another site than that at present occupied, but were much hampered for lack of funds. Mr. Birkin's generous gift will remove their difficulties, although some additions to the buildings and endowment will still be necessary. The Empress Frederick of Germany is expected to lay the foundation-stone in February, and a bazaar and presentation of purses will take place there for the benefit of the institution.

A great many large firms are sending edible commodities for the use of the wounded in the Transvaal. Amongst these are the manufacturers of Maggi's Consommé and Serrarallo's tonic wine.

Messrs. Burroughs and Wellcome have offered to supply the medical equipment of the "Maine" free of cost. Although the contributions in money are strictly confined to Americans, presents in kind will be accepted when suitable.

At the last meeting of the Liverpool Health Committee the Chairman stated that strict precautions to prevent the introduction of the plague were taken in conjunction with the sanitary administration of the Port and the Docks and Harbour Board.

The Executive Committee of the Paris International Congress of Medicine in 1900 have issued a list of sectional secretaries to whom the title and an abstract of any paper to be read must be sent. This list can be obtained from Dr. Garrod, 9, Chandos Street, W.; or D'Arcy Power, Esq., 10a, Chandos Street.

Sir William MacCormac sailed for Natal on Saturday accompanied by Mr. G. Makins. Mr. Treves, consulting surgeon to the London Hospital, and surgeon-in-ordinary to the Duke of York, follows immediately. Mr. Makins is surgeon at St. Thomas's Hospital. It would be difficult to have made a better selection of medical men to act as consulting surgeons to the troops in South Africa.

Italian medical men are still steadily pursuing the campaign against foreign practitioners carrying on practice without an Italian diploma. The Prime Minister is now inquiring of foreign Powers whether they will allow Italian doctors to practice with the diploma only of Italy in their respective countries. It is anticipated that, without evidence of reciprocity in their own country, foreign medical men will find themselves bound to secure an Italian diploma in order to pursue their practice.

The Local Government Board have received Dr. Reece's report on the sanitation of the Urban District of Aldershot. The matter is of special importance in connection with the health of the troops stationed there. The report is unfavourable to the sewage system; overcrowding and insanitation in the lodging-houses is pointed out; and the bye-laws relating to the keeping of horses are said to be disregarded. The large prevalence of diphtheria is commented upon, but no particular source of the evil is disclosed. Stricter vigilance and more thorough disinfection, notification, and isolation is advised.

In the *Nineteenth Century* for this month there is a useful account of the outbreak of plague in Oporto, and of the sanitary arrangements of the town. The account of the latter is of the greatest interest, showing as it does the existence of the same conditions of overcrowding, lack of light, and absence of the most elementary essentials of sanitation in those parts of Oporto which the disease has affected, and to which it has mostly clung, as have been found in other towns in which plague has obtained a considerable footing. The anticipations of the writer of the article in regard to the possibility of quickly eradicating the plague so long as these conditions continue are decidedly gloomy.

